# The effectiveness of hypertension management in China: a community-based intervention study

**DOI:** 10.1017/S1463423618000853

**Published:** 2019-07-16

**Authors:** Xiaoguo Zheng, Feng Xiao, Ruili Li, Delu Yin, Qianqian Xin, Huimin Yang, Tao Yin, Lihong Wang, Bowen Chen

**Affiliations:** 1 Department of Health Development, Capital Institute of Pediatrics, Beijing, China.; 2 Institute of Aging and Regenerative Medicine, Jinan University, Guangdong, China

**Keywords:** China, community-based intervention, effectiveness, hypertension management

## Abstract

**Aim:**

This study aimed to evaluate the effectiveness of hypertension management and analyse the factors associated with blood pressure reduction within China’s primary healthcare system.

**Background:**

Hypertension is one of the leading risk factors for global disease burden and is strongly associated with cardiovascular diseases. In China, hypertension is a serious public health problem, but few studies have evaluated the effectiveness of hypertension management in China’s primary healthcare system.

**Methods:**

The study sites were 24 primary healthcare institutions, selected using multistage stratified random sampling method. In each institution, hypertension patients aged at least 35 years who agreed to participate and had no disabilities or mental health problems were enrolled for hypertension management. Participants received comprehensive interventions in the primary healthcare system via a team. After a one-year intervention, data from 6575 hypertension patients were analysed to check the effectiveness of hypertension management and examined factors associated with hypertension control.

**Findings:**

There was an overall mean reduction of 4.5 mmHg in systolic blood pressure (SBP) and 1.9 mmHg in diastolic blood pressure (DBP). The blood pressure reduction after one year was greater in rural patients than in urban patients, 6.6 mmHg versus 3.4 mmHg for SBP and 2.6 mmHg versus 1.6 mmHg for DBP, respectively. The hypertension control rate also increased more in rural areas (22.1%) than in urban areas (10.6%) after the one-year intervention. Age, body mass index, region and being in an urban area had a significant negative association with the reduction of SBP (*P* < 0.05). Education level and baseline SBP showed a significant positive association (*P* < 0.05).

**Conclusions:**

Community-based hypertension management by general practitioners was feasible and effective. The effectiveness of hypertension management in rural areas was greater than in urban areas. Intervention strategies should pay more attention to patients in rural areas and western China.

## Introduction

Hypertension is a major risk factor for cardiovascular and cerebrovascular diseases and is a growing public health problem in China and globally (Katharina *et al*., 2003; McAlister *et al*., 2011). In 2010, 31.1% of the world’s adults had hypertension; 28.5% in high-income countries and 31.5% in low- and middle-income countries (Mills *et al*., 2016). In China, the prevalence of hypertension among adults increased from 5% in 1959 to 34% in 2010 (Jiasi, 1960; Longde, 2005; Hao *et al*., 2013). Hypertension imposes enormous health and economic burdens on China, with an estimated 153 million people currently having the condition (Pereira *et al*., 2009). The number of deaths each year from cardiovascular-related conditions as a result of hypertension is over 1.5 million in China (Yangfeng *et al*., 2008). The data indicate that hypertension control in China is far from satisfactory.

**Figure 1.**
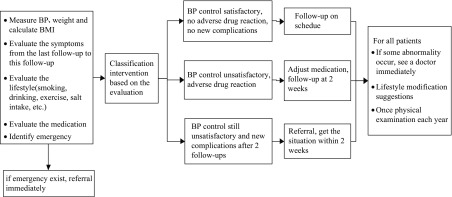
Interventions for hypertension patients

Poor management of hypertension leads to serious complications among patients, which places a heavy burden on the health system. It has been suggested that adequate control of hypertension in developing countries is achievable through community-based programmes and by encouraging the primary healthcare system to increase the focus on hypertension prevention and control (Rohina *et al*., 2008). In 2009, the Chinese government issued a strategy to provide 11 essential public health services to all residents through primary healthcare institutions such as township hospitals, village clinics and community health service centres. Hypertension management by general practitioners (GPs) in primary healthcare institutions was part of these services. GPs in China were similar to family physicians or GPs in other countries in that they provide comprehensive healthcare (covering chronic and acute diseases, preventive medicine and health education) (Qian *et al*., 2013). Hypertension interventions included health education, enhanced monitoring, family support, self-management, healthcare management changes and provider training (Zuxun *et al*., 2012). Hypertension patients received comprehensive interventions and obtained active and continuous services from GPs. Good follow-up of patients ensured that they were encouraged to improve their lifestyle, make rational use of drugs and reduce their blood pressure. During the management process, GPs were also able to detect other health problems and deal with them at an earlier stage. The management process is very clear and easy to operate (Figure 1). Through the one-year hypertension management programme, GPs could access professional training, and as a result the rural healthcare system was developed and strengthened.

Some research has been carried out since the implementation of hypertension management in primary healthcare centres, but there is little assessment of its efficacy in China. This study therefore aimed to investigate the effectiveness of hypertension management after a one-year intervention conducted as part of the national essential public health service via the primary healthcare system. The study also provided some insights for prevention and control of hypertension in this system.

## Materials and methods

### Study design

**Figure 2.**
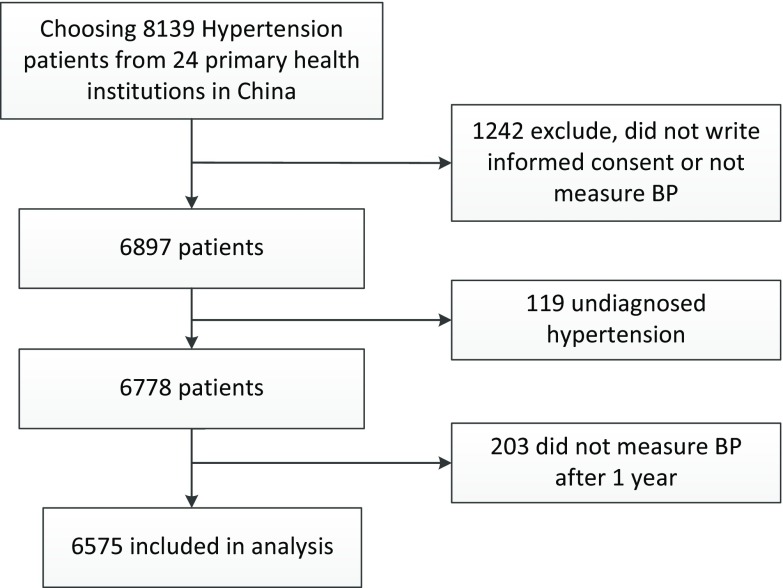
Flow chart showing patients from recruitment to inclusion in the study

A multistage stratified random sampling method was used to select the study sites. In the first stage, two provinces were selected from each region of China (east, central and west): Shandong and Zhejiang in the east, Anhui and Jiangxi in the central, and Shanxi and Guizhou in the west region of China. In the second stage, one urban and one rural area were randomly selected from each province, based on their social and economic development levels and populations. In the third stage, equal numbers of primary healthcare institutions in urban and rural areas were recruited, consisting of township hospitals in rural areas and community health centres in urban areas. Finally, 24 primary healthcare institutions were selected as the study sites. In each institution, hypertension patients aged at least 35 years who agreed to participate and had no disabilities or mental health problems were enrolled for hypertension management. The sample size in the study was powered to detect clinically meaningful differences in both systolic blood pressure (SBP) and diastolic blood pressure (DBP) of 2 mmHg after a one-year hypertension management programme. We assumed a normal approximation to compare two independent means. The sample size provided 95% power to detect differences, and the assumed SDs for SBP and DBP were both 10 mmHg. We used a two-sided α of 0.01 and a 75% response rate to estimate the sample size and enrolled 8139 hypertension patients. In total, 6778 patients finished the one-year management programme, and 203 did not have a blood pressure measurement after one year, giving a total of 6575 hypertension patients for analysis. A flow chart of patient from recruitment to inclusion was shown in Figure 2.

### Study intervention

Study participants received comprehensive interventions in the primary healthcare system via a team of GPs, nurses and public health doctors. The intervention included one physical examination and at least four face-to-face follow-ups with GPs in the course of the year. Further interventions were offered depending on the outcome of the follow-ups. Figure 1 shows the process. In all physical examinations, nurses collected data including height, weight, blood pressure and body mass index (BMI). Each follow-up included several essential interventions: (1) blood pressure measurement; (2) assessment of symptoms; (3) record of compliance with lifestyle modification and/or medication and (4) provision of prescriptions and further information as necessary.

All the participating GPs, nurses and public health doctors received training before the intervention. The training included hypertension screening, treatment and management, follow-up visits and health education.

### Data collection

At baseline information collection, GPs collected information from all patients about their socio-demographic characteristics, medication and whether they had diabetes or coronary heart disease. During the baseline physical examination, patient height and weight were measured, and BMI was calculated in kg/m^2^. Lean, normal weight, overweight and obese were defined in line with the Chinese standard criteria for weight for adults (WS/T 428–2013) as BMI < 18.5, 18.5 to < 24, 24 to < 28 and ≥ 28 kg/m^2^.

A trained nurse used a mercury sphygmomanometer to measure blood pressure three times on the patient’s right arm in a seated position after 5 min of rest. The mean of the three measurements was used for analysis. Patients were also advised to avoid alcohol, smoking, coffee and tea, and exercise for at least 30 min before their BP measurement. The Seventh Report of the Joint National Committee on Prevention, Detection, Evaluation, and Treatment of High Blood Pressure defined hypertension as SBP ≥ 140 mmHg and/or DBP ≥ 90 mm Hg, and/or use of antihypertensive medication (Chobanian *et al*., 2003). Hypertension was considered to be under control if blood pressure was < 140/90 mmHg.

All data used in the study were collected from the local study sites and stored in an online database. Information was collected from two physical examinations in October 2014 and in September 2015. The study was approved by the Ethical Committee of Research in the Capital Institute of Pediatrics. Written informed consent was obtained from each participant.

### Statistical analysis

Statistical analysis used SPSS for Windows, Version 15.0 (SPSS Inc., Chicago, IL, USA). Socio-demographic characteristics of the participants were analysed using descriptive statistics. The average reductions in SBP and DBP in different subgroups were given as the mean and 95% confidence interval (95% CI). The average reductions in SBP and DBP through the one-year programme were calculated using a paired *t*-test. Comparison among subgroups used one-way analysis of variance. Multiple linear regression analysis was used to evaluate the factors associated with reductions in SBP and DBP. In this model, the dependent variable was the reduction in SBP or DBP, and the independent variables were age (as a continuous variable), sex, education level, area (urban or rural), region (east, central or west), medication, reduction in BMI (as a continuous variable), and others. All statistical tests were two-tailed, with *P* < 0.05 considered statistically significant.

## Results

A total of 6575 hypertension patients (mean age: 64.6 ± 10.4 years) were eligible for analysis in the study. Of these, 40.7% were men, over 36% had received at least middle school education, 38.5% were overweight and 14.3% were obese. In total, 64.2% were from urban areas and 43.9%, 22.4% and 33.7% were from the eastern, central and western regions of China. Medically, 63.5% were on medication and 13.3% had diabetes as a comorbidity (see Table 1).

**Table 1.** Characteristics of the hypertension patients in the study

Characteristics	(*n* = 6575)	
	*n*	(%)
Sex		
Male	2679	40.7
Female	3896	59.3
Age (years)		
35–55	1094	16.6
55–65	2095	31.9
65–75	2214	33.7
≥ 75	1172	17.8
Marital status		
Married	5425	82.8
Unmarried	57	0.9
Divorced, separate, widowed	1067	16.3
Education level		
Illiterate or unknown	1776	27.0
Elementary	2391	36.4
Middle school	1619	24.6
High school and above	787	12.0
BMI (kg/m^2^)		
< 18.5	213	3.2
18.5–24.0	2885	43.9
24.0–28.0	2534	38.5
≥ 28.0	943	14.3
Region		
East	2887	43.9
Central	1475	22.4
West	2213	33.7
Community		
Urban	4220	64.2
Rural	2355	35.8
Treatment^a^		
Yes	4179	63.5
No	2396	36.6
Diabetic combination^b^		
Yes	876	13.3
No	5699	86.7

Note: Totals may vary because of missing values.

a Prescription medication treatment for hypertension.

b Hypertension and diabetes present in the same individual.

The mean SBPs before and after intervention were 145.6 ± 14.4 and 141.1 ± 13.4 mmHg. Mean DBP reduced from 92.3 ± 9.1 to 90.3 ± 8.3 mmHg after intervention. The hypertension control rate increased from 44.4% to 59.1%. The control rate was higher in urban than in rural areas both before and after intervention, but increased more in rural areas (22.1% vs 10.6% in urban areas) after the one-year intervention. The control rate rose in all three regions, from 57.3% to 68.9% (east), from 44.1% to 57.1% (central) and from 26.4% to 48.8% (west). Table 2 shows more results.

**Table 2.** The average blood pressure and control rate of hypertension pre- and post-intervention

Variable	SBP (Mean ± SD)		DBP (Mean ± SD)		Control rate (%)		
	Before	After	Before	After	Before	After	*P*
Total	145.6 ± 14.4	141.1 ± 13.4	92.2 ± 9.1	90.3 ± 8.3	44.4	59.1	<0.0001
Community							
Urban	142.7 ± 12.4	139.3 ± 11.9	90.2 ± 8.0	88.6 ± 7.4	55.9	66.5	< 0.0001
Rural	150.9 ± 16.1	144.3 ± 15.4	96.0 ± 9.7	93.4 ± 8.9	23.6	45.7	< 0.0001
Region							
East	141.8 ± 11.9	138.4 ± 11.8	89.7 ± 7.9	88.4 ± 7.3	57.3	68.9	< 0.0001
Central	146.1 ± 13.8	142.5 ± 13.0	92.1 ± 8.6	89.8 ± 8.0	44.1	57.1	< 0.0001
West	150.3 ± 14.4	143.5 ± 14.9	95.7 ± 9.7	93.2 ± 8.9	26.4	48.8	< 0.0001

SBP = systolic blood pressure; DBP = diastolic blood pressure.

Table 3 shows the reduction in blood pressure after the one-year intervention. The overall mean reduction was 4.6 mmHg (95% CI: 4.3–4.8 mmHg) in SBP and 1.9 mmHg (95% CI: 1.7–2.2 mmHg) in DBP. In the subgroup analysis, the reduction in SBP was higher among patients with higher levels of education, from the west and from rural regions (*P* < 0.01), and reduction in DBP was higher among younger patients, those with higher levels of education, and from the western region or rural areas (*P* < 0.01). There was no significant difference in SBP reduction between male and female patients (*P* > 0.05). However, the reduction in DBP was higher in men than women. Medication and concurrent diabetes showed no significant effect on the reduction in SBP and DBP (*P* > 0.05). There were significant differences between urban and rural areas in SBP and DBP (*P* < 0.01) [mean SBP and DBP reduction 6.6 mmHg (95% CI: 6.1–7.1 mmHg) vs 2.6 mmHg (95% CI: 2.2–2.9 mmHg)]. Similarly, region (east, central or west) was also significant for both SBP and DBP.

**Table 3.** Reduction in blood pressure after the one-year intervention (*n* = 6575)

Variable	SBP		DBP	
	Mean (95%CI)	*P*	Mean (95%CI)	*P*
Total	4.6 (4.3–4.8)	< 0.0001	1.9 (1.7–2.2)	< 0.0001
Sex				
Female	4.8 (4.4–5.2)	< 0.0001	2.2 (1.8–2.5)	< 0.0001
Male	4.3 (3.8–4.7)	< 0.0001	1.8 (1.5–2.1)	< 0.0001
Differ	0.5 (0.1–1.0)	0.067	1.0 (0.5–1.4)	< 0.0001
Age				
35–55 years	4.5 (3.9–5.1)	< 0.0001	2.7 (2.2–3.2)^a^	< 0.0001
55–65 years	4.5 (4.0–5.0)	< 0.0001	2.0 (1.7–2.4)	< 0.0001
65–75 years	4.9 (4.4–5.5)	< 0.0001	1.8 (1.4–2.2)	< 0.0001
≥ 75 years	4.0 (3.3–4.6)	< 0.0001	1.4 (0.9–1.9)	< 0.0001
Differ		0.14		0.00
Education level				
Illiterate or unknown	4.4 (3.8–4.9)	< 0.0001	1.4 (1.0–1.8)	< 0.0001
Elementary	3.3 (2.6–4.0)^b^	< 0.0001	1.7 (1.3–2.0)	< 0.0001
Middle school	4.7 (4.2–5.3)	< 0.0001	2.5 (2.1–2.9)	< 0.0001
High school and above	5.0 (4.5–5.5)	< 0.0001	2.8 (2.2–3.3)	< 0.0001
Differ		0.00		< 0.0001
BMI (kg/m^2^)				
< 18.5	4.4 (2.8–6.0)	< 0.0001	1.1 (−0.1–2.3)	< 0.0001
18.5–24.0	4.5 (4.1–4.9)	< 0.0001	1.9 (1.6–2.3)	< 0.0001
24.0–28.0	4.3 (3.9–4.8)	< 0.0001	1.8 (1.5–2.1)	< 0.0001
≥ 28.0	5.4 (4.6–6.1)	< 0.0001	2.5 (1.9–3.1)	< 0.0001
Differ		0.11		0.079
Region				
East	3.4 (3.0–3.8)	< 0.0001	1.3 (1.0–1.6)^c^	< 0.0001
Central	3.6 (3.0–4.1)	< 0.0001	2.4 (2.0–2.8)	< 0.0001
West	6.8 (6.3–7.3)^d^	< 0.0001	2.5 (2.2–2.9)	< 0.0001
Differ		< 0.0001		< 0.0001
Community				
Rural	6.6 (6.1–7.1)	< 0.0001	2.6 (2.2–2.9)	< 0.0001
Urban	3.4 (3.1–3.8)	< 0.0001	1.6 (1.3–1.9)	< 0.0001
Differ	3.2 (2.6–3.8)	< 0.0001	1.0 (0.5–1.4)	< 0.0001
Treatment				
Yes	4.7 (4.2–5.1)	< 0.0001	2.1 (1.8–2.4)	< 0.0001
No	4.5 (4.2–4.9)	< 0.0001	1.7 (1.3–2.0)	< 0.0001
Differ	0.2 (−0.4–0.7)	0.59	0.4(−0.0–0.8)	0.06
Diabetic combination				
Yes	4.5 (3.7–5.3)	< 0.0001	1.7 (1.1–2.2)	< 0.0001
No	4.6 (4.3–4.9)	< 0.0001	2.0 (1.8–2.2)	< 0.0001
Differ	−0.1 (−0.9–0.7)	0.80	−0.3 (−0.9–0.3)	0.28

SBP = systolic blood pressure; DBP = diastolic blood pressure; BMI = body mass index.

a There were significant differences in DBP reduction across age groups (33–55-year-old group compared with 55–65 years, 65–75 years and ≥ 75 years).

b The comparisons showed differences between elementary and illiterate or unknown, elementary and middle school, elementary and high school and above for SBP reduction.

c The comparisons between eastern and central region, and eastern and western region showed different DBP reduction.

d There were statistically significant differences in SBP reduction between western and eastern regions, and western and central regions.

Table 4 shows the results of the multiple linear regression analysis for the factors associated with blood pressure reduction. The results show that age, BMI, and living in an urban area and in eastern or central regions had a significant negative association with reduction in SBP (*P* < 0.05). However, having a level of education of at least middle school and baseline SBP had a positive association (*P* < 0.05). Age, BMI and living in the eastern region or an urban area were significantly associated with a reduction in DBP (*P* < 0.05), while a level of education of at least middle school and baseline DBP had a significant positive association with DBP reduction (*P* < 0.05). Sex and medication were not significantly associated with reductions in either SBP or DBP (*P* > 0.05).

**Table 4.** Results of the multiple linear regression for blood pressure (*n* = 6575)

Parameter	SBP		DBP	
	Estimate (SE)	*P*	Estimate (SE)	*P*
Intercept	−39.99 (1.88)	< 0.0001	−40.24 (1.34)	< 0.0001
Age (continuous)	−0.05 (0.01)	< 0.0001	−0.03 (0.01)	0.00
Education level				
Elementary	−0.22 (0.33)	0.51	0.05 (0.23)	0.87
Middle school	1.23 (0.39)	0.00	0.77 (0.27)	0.00
High school and above	1.11 (0.48)	0.02	0.95 (0.33)	0.01
Illiterate or unknown(ref.)	0		0	
BMI (continuous)	−0.09 (0.04)	0.01	−0.17 (0.03)	< 0.0001
Region				
East	−2.43 (0.68)	0.03	−0.95 (0.47)	0.04
Central	−3.21 (0.61)	0.00	−0.76 (0.42)	0.06
West (ref.)	0		0	
Community				
Urban	−2.41 (0.64)	< 0.0001	−3.15 (0.44)	< 0.0001
Rural (ref.)	0		0	
Baseline SBP	0.39 (0.01)	< 0.0001	_	_
Baseline DBP	_	_	0.59 (0.01)	< 0.0001

SBP = systolic blood pressure; DBP = diastolic blood pressure; BMI = body mass index.

## Discussion

The one-year hypertension intervention in this study generally reduced the SBP and DBP of the hypertension patients by a mean of 4.6 and 1.9 mmHg. The hypertension control rate increased from 44.4% to 59.1%. These results therefore show the intervention’s efficacy. There were several reasons for these results. First, the implementation of the national essential public health services required the GPs to establish health records for hypertension patients. Second, the primary healthcare centres offered annual health examinations and four sessions of face-to-face follow-up. This community-based hypertension management and support for blood pressure control is critical for hypertension patients. Some studies have shown that even a small reduction in blood pressure could markedly reduce the risk of coronary artery disease events (Staessen *et al*., 2003; Alireza *et al*., 2010). Based on those studies, our interventions are likely to be effective in reducing the risk for cardiovascular disease and its complications. The implementation of hypertension management is a powerful approach to reduce the blood pressure of hypertension patients and improve long-term health outcomes.

Health resources are unevenly allocated in China between urban and rural areas, and among the eastern, central and western regions. Patients in rural areas tend to be poorer than those in urban areas, have little professional medical contact and lack management strategies for chronic disease (Ross *et al*., 2006; Xiaohua *et al*., 2014). In rural areas, there are higher numbers of undetected, untreated and uncontrolled hypertension patients than in urban areas (Xinglin *et al*., 2010). In this study, the baseline blood pressure was different in urban and rural areas, and in eastern, central and western regions. The study found greater blood pressure reduction in patients in rural areas after one year. This may be because the hypertension management programme improved accessibility and allowed hypertension patients in the rural areas to obtain community-based prevention and control services, including knowledge about hypertension, and education about lifestyle modifications, as well as regular physician visits. There were also significant differences in blood pressure reduction by region, with the greatest reduction in the western region. The reason for this gap is probably similar to that for the urban–rural difference. Western areas in China are less developed than those in the eastern and central regions. The level of financial support for essential public health services has also historically been lower. Compared with patients with low education levels, patients with higher levels of education generally have more opportunities to acquire knowledge about hypertension, have greater health consciousness and develop healthier lifestyles. Higher levels of education were associated with greater reductions in blood pressure, a result consistent with previous studies (Gholamreza *et al*., 2013; Bin *et al*., 2014).

This study found that age and BMI were independently associated with better hypertension control. Hypertension is known to increase with age, so one possible explanation is that older people made more effective changes to their lifestyle, perhaps because of their poor self-perceived health status. Numerous studies have found that being overweight or obese is significantly associated with hypertension and poorer blood pressure control (Shuqiong *et al*., 2011; Xiujun *et al*., 2011), and our results were consistent with these. Hypertension patients who reported having concurrent diabetes mellitus have also been found likely to fail in controlling their hypertension (Jie *et al*., 2014). In our study, concurrent diabetes had no association with blood pressure control. This may be because these patients accounted for just 13.32% of the total, and the intervention time was just one year. Interestingly, a longer programme has previously been associated with a better blood pressure control rate, which emphasizes the importance of ongoing hypertension management (McInnis *et al*., 2008; Hirohide *et al*., 2010; Shelley *et al*., 2011).

In China, industrialization, urbanization, population aging and lifestyle changes are accelerating. The numbers of people with chronic diseases are also rapidly increasing each year. This poses a challenge for the primary healthcare system. This study found that a community-based health intervention for hypertension was associated with significantly improved blood pressure control. Our data will be helpful in designing and implementing future primary healthcare programmes for hypertension control and management.

## Conclusions

This study found that community-based hypertension management conducted by GPs was both feasible and effective. This suggests that implementing essential public health services could improve long-term health outcomes for chronic disease. Our data will be helpful in designing and implementing future primary healthcare programmes for hypertension control and management. The effectiveness of hypertension management in rural areas was greater than in urban areas. Intervention strategies should pay greater attention to patients in rural areas and western China.

## Limitations

The study had a few limitations. First, it did not have a comparative control group. This was because hypertension management had been improved in the national primary healthcare system. Second, we did not collect information about some important factors related to hypertension control, such as the duration and severity of hypertension, and comorbidities. We therefore could not calculate to what extent the intervention was responsible for improved blood pressure control. Third, our analysis focused on blood pressure reduction as the successful outcome of hypertension management in the primary healthcare system. We were unable to estimate the effects of factors such as lifestyle changes, medication, and hypertension complications, which would be useful information for public health policymakers. We also could not analyse the cost and cost-effectiveness of hypertension management, although this would also be useful for policymakers. Despite these limitations, the strengths of this study included regional representative data and an evidence-based rationale for control and management of hypertension at a population level.
